# Netupitant and palonosetron trigger NK_1_ receptor internalization in NG108-15 cells

**DOI:** 10.1007/s00221-014-4017-7

**Published:** 2014-06-27

**Authors:** Ajit G. Thomas, Marigo Stathis, Camilo Rojas, Barbara S. Slusher

**Affiliations:** 1 Brain Science Institute, Johns Hopkins University, 855 North Wolfe Street, Baltimore, MD 21205 USA; 2 Departments of Neurology, Psychiatry, and Neuroscience, Johns Hopkins University, Baltimore, MD 21205 USA; 3 Department of Molecular and Comparative Pathobiology, Johns Hopkins University, Baltimore, MD 21205 USA

**Keywords:** Chemotherapy-induced nausea and vomiting, NK_1_ receptor, 5-HT_3_ receptor, Netupitant, Palonosetron, Ondansetron

## Abstract

Current therapy for chemotherapy-induced nausea and vomiting includes the use of both 5-HT_3_ and NK_1_ receptor antagonists. Acute emesis has largely been alleviated with the use of 5-HT_3_ receptor antagonists, while an improvement in preventing delayed emesis has been achieved with NK_1_ receptor antagonists. Delayed emesis, however, remains a problem with a significant portion of cancer patients receiving highly emetogenic chemotherapy. Like other drugs in its class, palonosetron, a 5-HT_3_ receptor antagonist, has shown efficacy against acute emesis. However, palonosetron has also shown consistent improvement in the suppression of delayed emesis. Since both 5-HT_3_ and NK_1_ receptor antagonists are often simultaneously administered to patients, the question remains if palonosetron’s effect on delayed emesis would remain distinct when co-administered with an NK_1_ receptor antagonist. Recent mechanistic studies using NG108-15 cells have shown that palonosetron and netupitant, an NK_1_ receptor antagonist currently in phase 3 clinical trials, exhibited synergistic effects when inhibiting the substance P response. The present studies showed that both netupitant and palonosetron-induced NK_1_ receptor internalization in NG108-15 cells and that when used together receptor internalization was additive. Palonosetron-induced NK_1_ receptor internalization was dependent on the presence of the 5-HT_3_ receptor. Results provide a possible explanation for palonosetron’s enhancement of the inhibition of the SP response and suggest that the effect of palonosetron and NK_1_ receptor antagonists on prevention of delayed emesis could be additive.

## Introduction

Current therapy for the treatment of severe forms of chemotherapy-induced nausea and vomiting (CINV) caused by drugs like cisplatin includes the use of both 5-HT_3_ and NK_1_ receptor antagonists along with dexamethasone (Roila et al. 2010; Basch et al. 2011; Ettinger et al. 2012). Even though several neurotransmitters and their corresponding receptors have been associated with the pathogenesis of emesis (Darmani et al. 2009; Darmani and Ray 2009), acute CINV has been largely ameliorated with 5-HT_3_ receptor antagonists, while aprepitant, an NK_1_ receptor antagonist, was shown to improve overall antiemetic efficacy including delayed emesis (Hesketh et al. 2003; Darmani and Ray 2009; Feyer and Jordan 2011). Even though a majority of patients are fully protected against CINV by the use of these therapies, there are still a significant number of patients that experience nausea and delayed emesis, especially following highly or moderately emetogenic chemotherapies (Feyer and Jordan 2011). Netupitant is a potent and selective NK_1_ receptor antagonist (Rizzi et al. 2012) currently in phase III clinical trials in combination with a fixed dose of palonosetron for the prevention of CINV. Palonosetron is the only 5-HT_3_ receptor antagonist that has been found to be effective in both acute and delayed CINV after moderate emetogenic chemotherapy (Eisenberg et al. 2003; Gralla et al. 2003; Aapro et al. 2006; Saito et al. 2009). Since palonosetron does not bind to the NK_1_ receptor (Wong et al. 1995), the mechanism behind palonosetron’s unique efficacy among 5-HT_3_ receptor antagonists against nausea and delayed emesis has been puzzling. Mechanism of action studies have shown that unlike other 5-HT_3_ receptor antagonists, palonosetron exhibits allosteric interactions, positive cooperativity and persistent inhibition of receptor function; it also triggers receptor internalization and inhibits signaling crosstalk between 5-HT_3_ and NK_1_ receptors (Rojas et al. 2014). These molecular interactions may be responsible for palonosetron’s clinical profile. It is not clear however, if palonosetron’s suppression of delayed emesis would be synergistic, additive or obscured when used in combination with NK_1_ receptor antagonists. Recent studies using NG108-15 cells known to express both the 5-HT_3_ and NK_1_ receptors (Reiser and Hamprecht 1989; Emerit et al. 1993) showed that netupitant and palonosetron exhibit a synergistic effect in the prevention of the NK_1_ receptor response against its endogenous agonist substance P (Stathis et al. 2012). The results suggest that palonosetron’s effect is distinct in the presence of an NK_1_ receptor antagonist at least in NG108-15 cells. In the present study, we began to explore the potential mechanism behind the synergistic effect. We report that when used alone, both netupitant and palonosetron can trigger NK_1_ receptor internalization and that when used in combination their effect becomes additive. NK_1_ receptor internalization could be a result of the previously observed 5-HT_3_/NK_1_ receptor crosstalk inhibition by palonosetron (Rojas et al. 2010a).

## Methods

### Plasmid preparation and cell transfection

Plasmid preparation and cell transfection of HEK-293 cells to express NK_1_ receptors (NK_1_-HEK-293) cell have been described previously (Rojas et al. 2008).

### Antagonist concentrations

Unless otherwise noted, the concentration of each antagonist used was fivefold the K_d_ value reported in the literature to insure saturation of receptors. Thus, concentrations of netupitant, palonosetron and ondansetron were 5, 1 and 30 nM, respectively.

### Binding of [^3^H]-netupitant after incubation of cells with unlabeled antagonists

[^3^H]-Netupitant binding to cells after preincubation with different antagonists was measured as outlined previously (Rojas et al. 2008). Briefly, NG108-15 cells or NK_1_-HEK-293 cells were pretreated with antagonist(s) or saline (control) for 24 h. Subsequent to each treatment, antagonists were removed, and fresh growth media were added to the cells; 2 h later, cells were incubated in fresh HEPES-buffered saline at room temperature for an additional 30 min. The buffer was removed and cells incubated with [^3^H]-netupitant (5 nM) for 40 min at room temperature. At the end of the incubation period, media were removed and the cells were washed with ice-cold buffer. Following the wash, cells were lysed into 200 µl of fresh ice-cold buffer and the radioactivity associated with the cells was measured using a scintillation counter. Student’s *t* test was used for statistical analyses of the results.

### Dissociation of antagonists from cells

NG108-15 cells were incubated with [^3^H]-netupitant ± palonosetron or ondansetron for 24 h. At the end of this incubation, antagonist-containing media were replaced with prewarmed HEPES-buffered saline containing excess unlabeled netupitant (5 µM) and dissociation of [^3^H]-netupitant at 37 °C was followed at 0, 2.5, 5, 7.5, 15, 30, 60 and 120 min. After removing medium, cells were scraped into 200 µl of fresh ice-cold buffer, and the radioactivity present in the scraped material at each time point was measured using a scintillation counter. Student’s *t* test was used for statistical analyses of the results.

### Dissociation of antagonists from cell-free membranes

Preparation of cell-free membranes and kinetic dissociation experiments using cell-free membranes have been described previously (Wong et al. 1995; Rojas et al. 2008). Briefly, the association phase was conducted in a 96-well glass plate (Zinsser NA, Northridge, CA) by incubating NG108-15 cell membranes prepared from ~100,000 cells with [^3^H]-netupitant ± palonosetron or ondansetron in Tris-Krebs buffer (pH 7.4 at 37 °C) for 90 min at 37 °C. The dissociation phase was then initiated by addition of excess unlabeled netupitant (1 μM). The amount of [^3^H]-netupitant bound to the receptor was measured at various times during the first hour after addition of displacer. Prism (GraphPad Software Inc, San Diego, CA) was used to obtain half-life values.

### Acid treatment

The acid treatment protocol was based on published methodology (Haigler et al. 1980). NG108-15 cells were incubated with [^3^H]-netupitant ± palonosetron or ondansetron for 24 h. At the end of this period, media were removed and cells were incubated with saline (0.5 M NaCl) containing acetic acid (0.2 M, pH 2.5) for 6 min on ice. Acid denaturation of cell surface proteins was terminated with the addition of one volume of ice-cold HEPES-buffered saline (pH 7.4). Cells were then washed once with the same buffer. Radioactivity present in the cells was measured with a scintillation counter, and percent radioactivity in the cell fraction was calculated. Radioactivity present in washes was also measured to confirm that the radioactivity recovery was close to 100 % in each case. Student’s *t* test was used for statistical analysis of the results.

### Protease treatment

The protease treatment protocol was adapted from the literature (Simantov and Sachs 1973). Briefly, NG108-15 cells were incubated with [^3^H]-netupitant ± palonosetron or ondansetron for 24 h. At the end of this period, media were removed and cells were incubated with HEPES-buffered saline containing trypsin (2.5 mg/ml) for 5 min at 37 °C. Digestion by trypsin was terminated by washing cells twice with ice-cold HEPES-buffered saline containing limabean trypsin inhibitor (50 µg/ml). Radioactivity present in each wash and in the cells was determined with a scintillation counter, and percent radioactivity in the cell fraction was calculated. A control experiment was carried out to measure dissociation of antagonists from cells, in the absence of proteases, under similar experimental conditions. Student’s *t* test was used for statistical analysis.

## Results

### Preincubation of NG108-15 cells with netupitant plus palonosetron additively reduced [^3^H]-netupitant binding

**Fig. 1:**
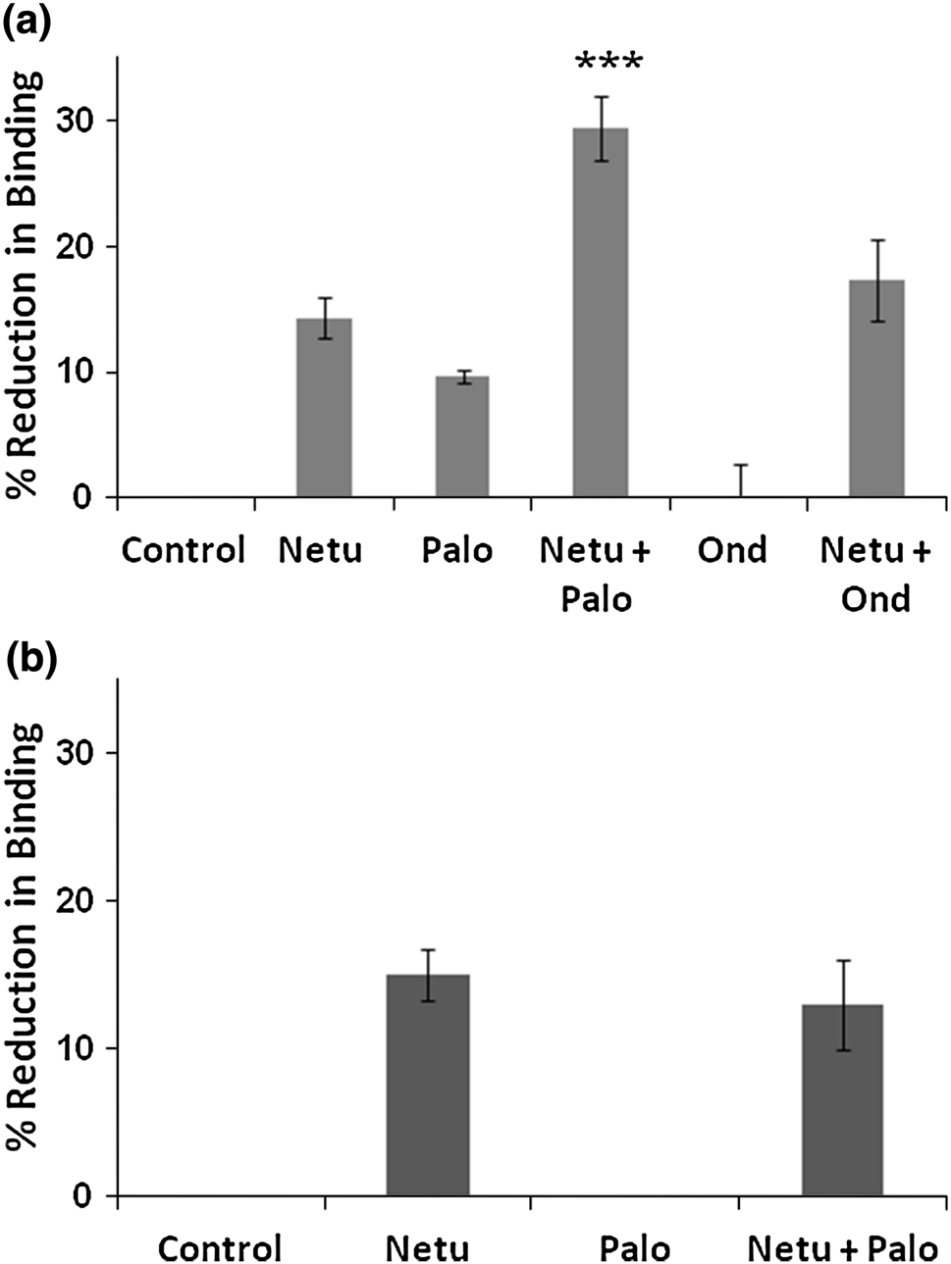
Reduction in binding of [^3^H] netupitant after incubation of cells with NK_1_ and 5-HT_3_ receptor antagonists. Reduction in binding in **a** NG108-15 cells expressing both NK_1_ and 5-HT_3_ receptors and **b** NK_1_-HEK-293 cells expressing NK_1_ receptors only—cells were preincubated ± antagonist (s) for 24 h as indicated. Netu: netupitant (5 nM); Palo: palonosetron (1 nM); Netu + Palo: netupitant (5 nM) plus palonosetron (1 nM); Ond: ondansetron (30 nM); Netu + Ond: netupitant (5 nM) plus ondansetron (30 nM). Cells were washed to remove the antagonists and subsequently incubated with [^3^H] netupitant for 40 min at room temperature. At the end of the incubation period, unbound [^3^H] netupitant was removed, and the radioactivity associated with the cells was measured (methods). Radioactivity associated with control cells was normalized to 100 %. Percent binding with respect to control cells for each treatment was subtracted from 100 to obtain the reduction in binding shown on the *y*-axis. *Error bars* correspond to standard deviation of three independent experiments run in triplicate. ***p < 0.001 when compared to netupitant; student’s *t* test was used for statistical analysis

When NG108-15 cells expressing both 5-HT_3_ and NK_1_ receptors were pretreated with either netupitant (5 nM) or palonosetron (1 nM), subsequent binding of [^3^H]-netupitant was reduced, respectively, by 14 ± 0.7 and 10 ± 0.4 % compared with binding by control cells that had not been preincubated with antagonist. Further, when cells were preincubated with the two antagonists, reduction in binding was additive: 29 ± 1.7 % (Fig. 1a). In contrast, when NG108-15 cells were pretreated with ondansetron (30 nM), there was no reduction in binding compared with control cells (0 ± 1.4 %). Moreover, when ondansetron was preincubated with netupitant, reduction in binding was the same as when using netupitant only (17 ± 2 %) (Fig. 1a).

Interestingly, when using NK_1_-HEK-293 cells that express the NK_1_ receptor but not the 5-HT_3_ receptor, preincubation with palonosetron did not reduce subsequent [^3^H]-netupitant binding (Fig. 1b). However, preincubation with netupitant reduced [^3^H]-netupitant binding by 15 ± 1 %, but when palonosetron and netupitant were preincubated together with NK_1_-HEK293 cells, reduction in binding was the same as when using netupitant only (13 ± 2 %) (Fig. 1b).

### Acid treatment of NG108-15 cells preincubated with [^3^H]-netupitant plus palonosetron showed additive association of [^3^H]-netupitant with cells

When cells are incubated with [^3^H] ligand that binds to a receptor followed by removal of media and limited acid treatment, the acid partially disrupts cell membranes. This disruption removes [^3^H] ligand that could be buried within the cell membrane leaving only radiolabeled material that is truly inside cells. The presence of radiolabeled material inside cells after acid treatment is indicative of receptor internalization (Haigler et al. 1980; Rojas et al. 2010b). Accordingly, when NG108-15 cells were incubated with [^3^H]-netupitant followed by limited exposure to acetic acid, the percent of [^3^H]-netupitant associated with cells was 14 ± 0.5 % compared to when cells were only washed with cold buffer, but not treated with acid. When both [^3^H]-netupitant and palonosetron were used, the percent of [^3^H]-netupitant associated with cells became additive: 28 ± 1.4 % (Table 1). In contrast, the use of ondansetron with [^3^H]-netupitant did not increase the amount of radiolabel associated with the cells (13 ± 0.1 %) (Table 1).

**Table 1:** Acid Treatment—NG108-15 cells were incubated for 24 h with [^3^H]-netupitant (5 nM), [^3^H]-netupitant (5 nM) plus palonosetron (1 nM) or [^3^H]-netupitant (5 nM) plus ondansetron (30 nM). After incubation, media were removed and cells were exposed to acetic acid pH 2.5 for 6 min on ice or to saline containing trypsin for 5 min at 37 °C. Cells were washed twice to remove radioactivity released into buffer and radioactivity remaining with cells was measured. Values listed are percent of [^3^H]-netupitant remaining with cells compared to cells that were washed with cold buffer but did not undergo acid or protease treatment. Data are the average of at least three independent determinations run in triplicate ± standard deviation

	Percent [^3^H]-netupitant remaining with cells after acid treatment	Percent [^3^H]-netupitant remaining with cells after protease treatment
[^3^H]Netupitant	14 ± 0.5	14 ± 0.8
[^3^H]-Netupitant plus palonosetron	28 ± 1.4***	27 ± 0.1***
[^3^H]Netupitant plus ondansetron	13 ± 0.1	13 ± 1

*** *p* < 0.001 when compared to [^3^H]-netupitant

### Protease treatment of NG108-15 cells preincubated with [^3^H]-netupitant plus palonosetron showed additive association of [^3^H]-netupitant with cells

When cells are incubated with [^3^H] ligand that binds to a receptor followed by removal of media and limited protease (trypsin) treatment, the protease partially hydrolyzes proteins present on the cell membranes. This partial hydrolysis denatures the proteins and removes [^3^H]-netupitant that could be pseudo irreversibly bound to the receptor within the cell membrane. As a result, any [^3^H]-netupitant that is associated with cells is [^3^H]-netupitant that is truly inside cells. The presence of radiolabeled material inside cells after protease treatment is indicative of receptor internalization (Simantov and Sachs 1973; Rojas et al. 2010b). Accordingly, when NG108-15 cells were incubated with [^3^H]-netupitant followed by limited exposure to trypsin, the percent of [^3^H]-netupitant associated with cells was 14 ± 0.8 % compared to when cells were only washed with cold buffer but not treated with trypsin. When both [^3^H]-netupitant and palonosetron were used, the percent of [^3^H]-netupitant associated with cells became additive: 27 ± 0.1 % (Table 1). In contrast, the use of ondansetron with [^3^H]-netupitant did not increase the amount of radiolabel associated with the cells (13 ± 1 %) (Table 1).

### The extent of [^3^H]-netupitant associated with cells is increased in the presence of palonosetron, but not ondansetron

**Fig. 2:**
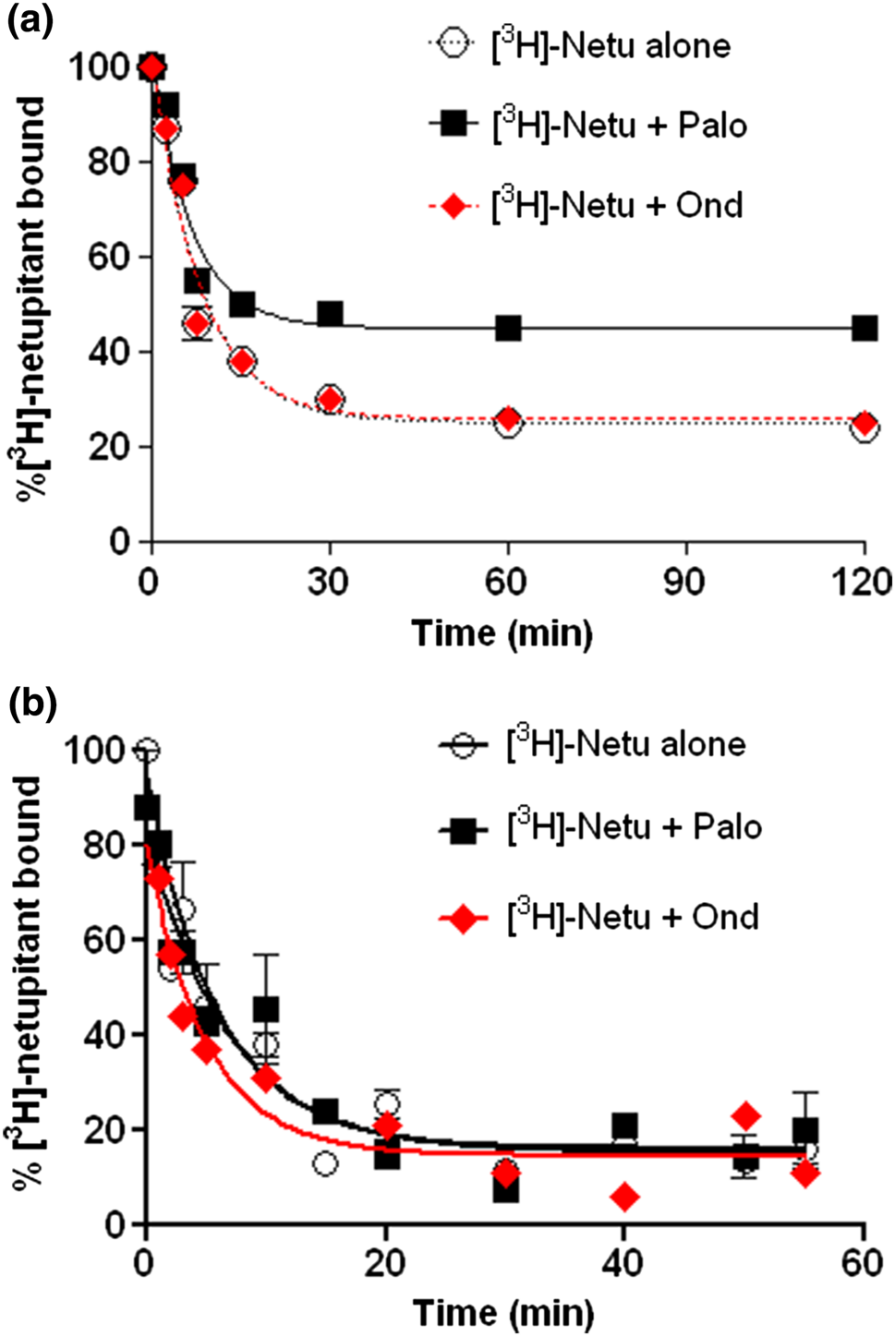
Dissociation of [^3^H]-netupitant ± palonosetron or ondansetron from NG108-15 cells (**a**) and cell-free membranes (**b**). Cells were preincubated for 24 h with [^3^H]-netupitant (5 nM), [^3^H]-netupitant (5 nM) plus palonosetron (1 nM) or [^3^H]-netupitant (5 nM) plus ondansetron (30 nM). At the end of this incubation, antagonist-containing media were replaced with prewarmed HEPES-buffered saline containing excess unlabeled netupitant (5 µM) and dissociation of [^3^H]-netupitant at 37 °C was followed at different times as shown. After removing medium, cells were scraped into 200 µl of fresh ice-cold buffer, and the radioactivity present in the scraped material at each time point was measured using a scintillation counter. When using cell-free membranes the association phase was carried out for 90 min (24 h gave same results) at 37 °C. The dissociation phase was then initiated by addition of excess unlabeled netupitant (1 μM). The amount of [^3^H]-netupitant bound to the receptor was measured at various times during the first hour after addition of displacer. Prism (GraphPad Software Inc, San Diego, CA) was used to obtain half-life values

If [^3^H]-netupitant goes inside cells as part of a [NK_1_ receptor-[^3^H]-netupitant] complex, it would not be available for dissociation. Accordingly, we studied dissociation of [^3^H]-netupitant from NG108-15 cells after allocating time for internalization (24 h) ± palonosetron or ondansetron. The percent of [^3^H]-netupitant that remained associated with cells after 1 h under dissociation conditions was 24 ± 2 % of the radioactive material associated with cells at the beginning of dissociation (Fig. 2a and Table 2). When cells were preincubated with [^3^H]-netupitant plus palonosetron, the percent of [^3^H]-netupitant that remained associated with cells increased almost twofold to 45 ± 0.6 % (Fig. 2a and Table 2), an indication of increased [NK_1_ receptor-[^3^H]-netupitant] complex internalization. In contrast, when cells were preincubated with [^3^H]-netupitant plus ondansetron, the percent of [^3^H]-netupitant that remained associated with cells remained the same as when preincubating with [^3^H]-netupitant alone: 25 ± 0.2 % (Fig. 2a and Table 2). Dissociation from cells in every case reached a plateau at 1 h and remained the same at 2 h (Fig. 2). The half-life of dissociation of [^3^H]-netupitant from cells was the same within error for the three treatments (~5 min) (Table 2).

**Table 2:** Rate and extent of dissociation in cells and cell-free membranes—values of half lives correspond to the traces in Fig. 2. Graph Pad PRISM^®^ was used to obtain half lives using the best fit of a single phase exponential decay. Data are the average of four experiments. Errors correspond to ± SEM

Treatment	%[^3^H]-Netupitant associated with cells after dissociation levels off (120 min)	Half-life of [^3^H]- Netupitant bound to cells (min)	Half-life of [^3^H]- Netupitant bound to cell-free membranes (min)
[^3^H]- Netupitant	24 ± 2	5.7 ± 0.3 (*n* = 3)	4.7 ± 0.2 (*n* = 2)
[^3^H]- Netupitant + palonosetron	45 ± 0.6***	4.6 ± 0.2 (*n* = 3)	4.3 ± 0.8 (*n* = 3)
[^3^H]- Netupitant + ondansetron	25 ± 0.2	5.6 ± 0.11 (*n* = 3)	3.5 (*n* = 1)

*** *p* < 0.001 when compared to [^3^H]-netupitant

### The extent of [^3^H]-netupitant dissociation from cell-free membranes is the same in the presence or absence of palonosetron or ondansetron

If cell-free membranes are used in dissociation experiments, no internalization into cells is possible, and the extent of dissociation should be the same for all antagonists. Accordingly, when cell-free membranes isolated from NG108-15 cells were incubated with [^3^H]-netupitant ± palonosetron or ondansetron, the extent of dissociation was the same for all three treatments (Fig. 2b). As expected, dissociation of [^3^H]-netupitant was close to 100 % except for some residual non-specific binding of netupitant (Fig. 2b). The half-life of dissociation of [^3^H]-netupitant from cell membranes was the same within experimental error for all three treatments (~4 min) (Table 2).

## Discussion

Previous studies have shown that palonosetron, in contrast to ondansetron and granisetron, causes 5-HT_3_ receptor internalization and inhibits SP-mediated responses in vitro and in vivo possibly as a result of inhibition of 5-HT_3_/NK_1_ receptor cross talk (Rojas et al. 2014). These results gave a tentative rationale for palonosetron’s improved ability among 5-HT_3_ receptor antagonists to prevent nausea and delayed emesis after moderate emetogenic chemotherapy. In any case, given the inhibition of delayed emesis by NK_1_ receptor antagonists, it is not clear if palonosetron’s inhibition of delayed emesis would make any difference if co-administered with an NK_1_ receptor antagonist. Recent studies using NG108-15 cells that express both the NK_1_ and 5-HT_3_ receptors showed synergistic inhibition of the substance P response by palonosetron and netupitant (Stathis et al. 2012). The mechanism of this combined inhibition of the SP response is not known. One possible explanation is that in addition to direct antagonism of the NK_1_ receptor by netupitant, palonosetron could exert an indirect regulatory effect on the NK_1_ receptor after antagonizing the 5-HT_3_ receptor. A case of a ligand binding to one receptor and triggering internalization of another receptor has been reported previously: NMDA binding to the NMDA receptor triggers internalization of the GABA_B_ receptor (Guetg et al. 2010). We present three independent lines of evidence to show that NK_1_ receptor internalization can be triggered by netupitant or palonosetron, but not by ondansetron. We also show that NK_1_ receptor internalization by netupitant and palonosetron is additive and that palonosetron-triggered NK_1_ receptor internalization requires the presence of 5-HT_3_ receptors. First, binding of [^3^H]-netupitant was reduced after prior exposure to netupitant or palonosetron, but not ondansetron (Fig. 1). Second, [^3^H]-netupitant remained with cells following incubation with [^3^H]-netupitant ± palonosetron and subsequent acid or protease treatments. When palonosetron was present, [^3^H]-netupitant association with cells was increased. In contrast, when using ondansetron, the amount of [^3^H]-netupitant association with cells was the same as when using [^3^H]-netupitant alone (Table 1). Finally, dissociation of [^3^H]-netupitant from cells was significantly reduced in the presence of palonosetron, but not ondansetron. In contrast, when using cell-free membranes where no receptor internalization can occur, the extent of dissociation of [^3^H]-netupitant was the same ± palonosetron or ondansetron (Fig. 2).

**Fig. 3:**
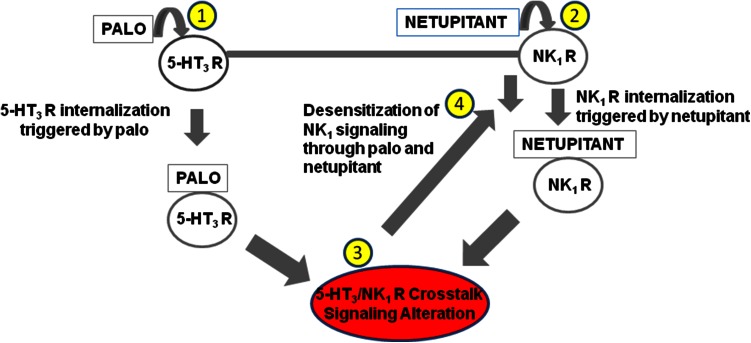
Effects of netupitant and palonosetron in NG108-15 cells—Receptor internalization can be induced through direct binding of palonosetron to the 5-HT_3_ receptor (*1*) and of netupitant to the NK_1_ receptor (*2*). Internalization of either receptor could lead to alterations in receptor signaling cross talk (*3*). NK_1_ receptor internalization would result in lower NK_1_ receptor density at the cell surface that in turn desensitizes NK_1_ signaling (*4*). NK_1_ receptor internalization can be induced by netupitant (directly and possibly indirectly) or by palonosetron (indirectly)

NK_1_ receptor internalization by netupitant and by palonosetron provides a rationale on how these antagonists could be acting in concert to help prevent NK_1_ receptor activation by substance P in NG108-15 cells (Stathis et al. 2012). This rationale is illustrated in Fig. 3. Previously, we have shown that palonosetron can induce 5-HT_3_ receptor internalization (Rojas et al. 2010b). Further, work in other laboratories has shown that there is receptor cross talk between 5-HT_3_ and NK_1_ receptor pathways (Minami et al. 2001; Hu et al. 2004). It is possible that 5-HT_3_ receptor internalization induced by palonosetron alters this receptor cross talk, resulting in NK_1_ receptor internalization. In parallel, netupitant also triggers NK_1_ receptor internalization. Both events lead to reduced density of NK_1_ receptors on the cell surface; since palonosetron and netupitant trigger receptor internalization only partially, it is possible for NK_1_ internalization to be additive and inhibition of downstream signaling like substance P-induced calcium ion mobilization to be synergistic (Stathis et al. 2012). We are currently exploring effects on phosphorylation by these antagonists in an effort to further elucidate the effects of these drugs on the crossroads of these two signaling systems.

It is important to remember that NG108-15 cells are a rodent-derived cell model used to explore the potential mechanism of action on delayed emesis when using palonosetron and netupitant in humans. Consequently, the results obtained in these studies are only a tentative explanation of the more complex mechanism in humans. While the use of 5-HT_3_ and NK_1_ rodent receptors has been useful in the early characterization of palonosetron (Wong et al. 1995) and netupitant (Rizzi et al. 2012) before they went to the clinic, there are differences between the receptors in the two species that could be relevant to their mechanism of action in the two species. For example, the affinity of rodent NK_1_ receptor antagonists is lower than in humans (Tattersall et al. 2000; Rizzi et al. 2012). In addition, even though both rodents and humans exhibit 5-HT_3A_ and 5-HT_3B_ receptor subunits, humans also have 5-HT_3C-E_ subunits that could conceivably alter the response to the antagonists (Lummis 2012).

In summary, three independent lines of evidence indicate that both netupitant and palonosetron can trigger NK_1_ receptor internalization in NG108-15 cells. Netupitant exerts its effect through direct binding, whereas palonosetron does it through a 5-HT_3_ receptor-mediated effect. When both antagonists are used, receptor internalization becomes additive. 5-HT_3_ and NK_1_ receptor internalization by both netupitant and palonosetron provide additional information in the effort to arrive to a mechanistic explanation of the additive and synergistic effects on the NK_1_ receptor antagonism when a combination of netupitant and palonosetron is used in these cells.
